# Bleeding detection in wireless capsule endoscopy videos — Color versus texture features

**DOI:** 10.1002/acm2.12662

**Published:** 2019-06-28

**Authors:** Konstantin Pogorelov, Shipra Suman, Fawnizu Azmadi Hussin, Aamir Saeed Malik, Olga Ostroukhova, Michael Riegler, Pål Halvorsen, Shiaw Hooi Ho, Khean‐Lee Goh

**Affiliations:** ^1^ Department of Communication Systems Simula Research Laboratory Fornebu Norway; ^2^ Center of Intelligent Signal & Imaging Research Group Universiti Teknologi PETRONAS Tronoh Perak Malaysia; ^3^ Research Institute of Multiprocessor Computation Systems n.a. A.V. Kalyaev Russia; ^4^ Department of Medicine University of Malaya Medical Center Kuala Lumpur Malaysia

**Keywords:** bleeding detection, color feature, machine learning, texture feature, wireless capsule endoscopy

## Abstract

Wireless capsule endoscopy (WCE) is an effective technology that can be used to make a gastrointestinal (GI) tract diagnosis of various lesions and abnormalities. Due to a long time required to pass through the GI tract, the resulting WCE data stream contains a large number of frames which leads to a tedious job for clinical experts to perform a visual check of each and every frame of a complete patient’s video footage. In this paper, an automated technique for bleeding detection based on color and texture features is proposed. The approach combines the color information which is an essential feature for initial detection of frame with bleeding. Additionally, it uses the texture which plays an important role to extract more information from the lesion captured in the frames and allows the system to distinguish finely between borderline cases. The detection algorithm utilizes machine‐learning‐based classification methods, and it can efficiently distinguish between bleeding and nonbleeding frames and perform pixel‐level segmentation of bleeding areas in WCE frames. The performed experimental studies demonstrate the performance of the proposed bleeding detection method in terms of detection accuracy, where we are at least as good as the state‐of‐the‐art approaches. In this research, we have conducted a broad comparison of a number of different state‐of‐the‐art features and classification methods that allows building an efficient and flexible WCE video processing system.

## 1 INTRODUCTION

Bleeding in the GI tract may be an indication of various abnormalities such as ulcerative colitis (UC), vascular tumors, and inflammatory disease.^1^ The standard diagnosis procedure is the manual inspection of the entire GI tract performed by an experienced clinician in order to detect bleeding as one of the most common abnormalities which may indicate a disease. Traditional endoscopy techniques such as sonde and push enteroscopy are painful and risky procedures for the patients as it can tear intestinal walls in case of severe medical conditions. Also, they have limitations to reach and visualize the small intestine.^2^, ^3^ The wireless capsule endoscopy (WCE) technology, which made its debut around the year 2000, uses a wireless electronic device^4^ that captures images or videos of the entire GI tract. The capsule, shaped like a normal pill, can be swallowed by the patient in the presence of clinical experts without any discomfort. Unlike conventional endoscopy procedures, it explores the whole GI tract of the patient without any pain, sedation, and air insufflation. The Food and Drug Administration (FDA) approved the use of WCE in 2001 as a medical tool to examine the mucosa of the stomach and small intestine in order to detect various abnormalities and diseases. Until now, the WCE technology has assisted more than 1.6 million patients worldwide.

**Figure 1:**
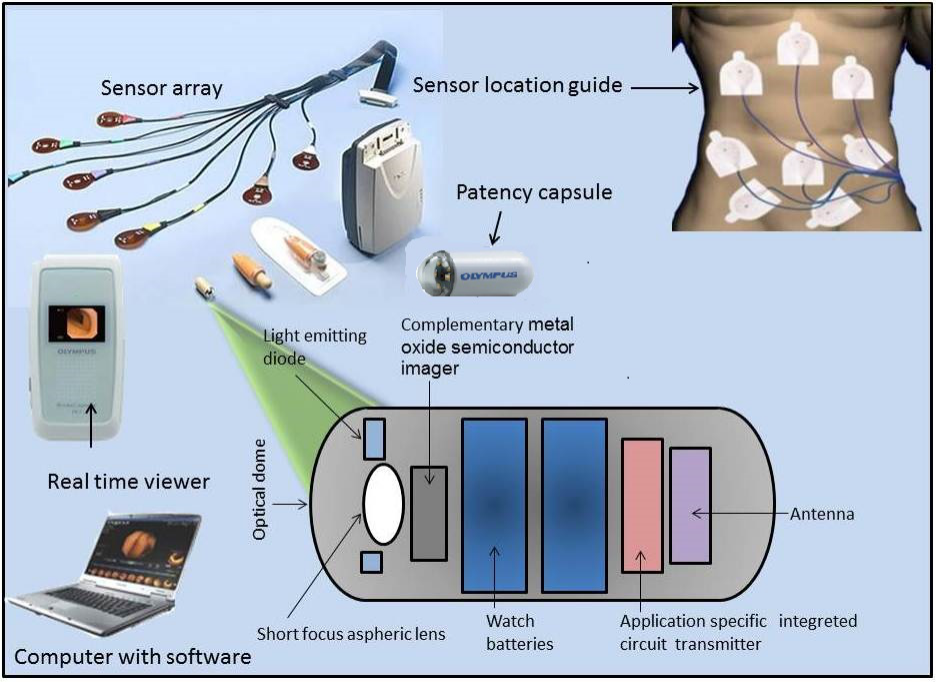
Composition of WCE and data acquisition setup.^5^

Figure 1 shows the typical internal components of a WCE. Modern WCEs are pill‐shaped (26 mm × 11 mm) devices, and they consist of the light sources, a short focal length charge‐coupled device (CCD) camera, a radio frequency transmitter, a battery‐based power supply, and a few other electronic components. Once a patient swallows the capsule, the WCE starts capturing frames with 1–30 frames per second (FPS), depending on the device type and its purpose, and the frames are sent wirelessly to the recorder unit. This process usually takes 8–10 h before the WCE’s battery is drained. During this time, the WCE has produced around 50,000–80,000 frames for each patient. The captured video allows clinicians to diagnose and detect ulcers, tumors, bleedings, and other lesions within the GI tract later offline to make diagnostic decisions. Although the WCE technology has many advantages, there is still room for research. For example, currently, it is tough for the clinicians to inspect the whole set of 50,000 and more frames to locate a disease. They might miss the disease at the early stage due to visual fatigue and small size of the lesion area. A software was developed by Given Imaging, which aims to detect active blood automatically, but the sensitivity and specificity are reported very low.^6^

A new method is proposed in this paper, based on morphological operations and a machine‐learning‐based classification including a support vector machine (SVM) to differentiate between normal and abnormal frames for bleeding findings. As color and texture are the main features to explore bleeding frame candidates, this paper is focusing on color detection in the red‐green‐blue (RGB) color space and various texture features. Experimental analysis depicts that this method is capable of performing bleeding detection with the performance achieved at least as good as the state‐of‐the‐art techniques. At the same time, this paper provides the broad comparison and analysis of the different state‐of‐the‐art features and classification methods in terms of their usability for building the efficient and flexible WCE video processing systems. The remainder of this paper is organized as follows: Section 2 provides a short survey of the related works found in the literature. Section 3 describes our methodology and the proposed algorithm. Results and discussions are presented in section 4. Finally, in section 5, we present our conclusions and provide directions for future work.

## 2 RELATED WORK

**Figure 2:**
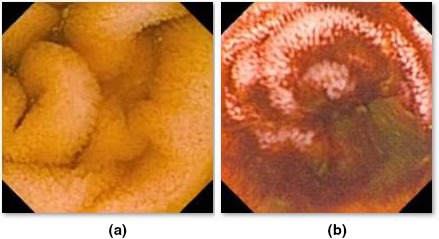
Wireless capsule endoscopy frame samples: normal GI mucosa (a) and active bleeding (b).

Bleeding is very a common abnormality found in the GI tract. Many researchers have contributed to detecting this with high‐performance classifiers. It is crucial to detect bleeding at an early age since it is a precursor for inflammatory bowel diseases such as Crohn’s disease and UC. Figures 2(a) and 2(b) show the normal mucosa and bleeding, respectively. Bleeding are not limited to the stomach, but in fact, they can occur anywhere in the whole GI tract,^7^ and they can be considered as a common anomaly detected by WCEs often defined as "bleeding of unknown origin that recurs or persist or is visible after an upper endoscopy and/or negative endoscopy result".^8^ The primary challenge is that blood spot and residual traces do not have any typical shape and texture, and the color of blood might vary from light red to dark intense red and brown, which makes the blood challenging to differentiate from the intestinal content or other objects present in the intestine. This diversity of color might depend on the position of the camera capsule, the bleeding timing^9^ and the surrounding condition of the intestinal content.^10^ Bleeding is not a single pathology, and it may be caused by a variety of small intestinal diseases, such as angiodysplasia, open wounds, ulcer, vascular lesions, tumors, and Crohn's disease. Both color and texture features have been used to discriminate pathology, and some related works are discussed in this section.

Baopu Li^11^ incorporated an intelligent system to detect a bleeding region in WCE videos using chrominance‐moments‐based texture. Mathew and Gopi^12^ have presented a method of discrimination between bleeding and nonbleeding frames using a contourlet transform with two levels of decomposition for color and texture features into coarse band and sub‐bands. A rotation invariant Local binary pattern is applied on coarse band and sub‐bands. Liu and Gan^13^ have designed an algorithm using a joint diagonalization principal component analysis (PCA) combined with the color coherence vector (CCV) where no iterations, approximations, and inverting procedures are required. This method overcomes the problem of PCA and the “curse of dimensionality” of the original asymptotic PCA. Tuba et al.^14^ proposed an algorithm for automatic segmentation for bleeding detection in WCE frames in HIS color space using the intensity channel for extracting texture features. They calculated a histogram for uniform LBP with 8 x 8 regions in terms of mean, variance, entropy, kurtosis, skewness, and energy.

Furthermore, Liu and Gu^15^ used covariance wavelet transform to discriminate between normal and abnormal tissue. Color information is extracted from the most used color spaces RGB, CIE Lab, XYZ, HSI, K‐L, and HSV. The texture feature is extracted using a discrete wavelet transform for multi‐resolution analysis. The color wavelet covariance features were obtained in each color channel of the frame. Piotr Szczypinski^16^ introduced an ANOVA using the F‐statics measure and the sequential floating forward search to classify various abnormalities such as bleeding and ulcer (excessive ulcer) for color and texture features. Yeh and Wu^17^ also proposed a novel method for detecting bleeding and ulcers in WCE frames. RGB, HSV, and CCV are used to compute color features. The frames were transformed into grayscale frames that are binarized on a predefined threshold. Yuan and Bapou Li^18^ introduced a new method for WCE frame classification with various abnormalities such as bleeding, ulcers, and polyps. They first build up bag‐of‐visual‐words by extracting scale‐invariant feature transform features from normal and abnormal frames, followed by a novel coding method based on saliency and adaptive locality constrained linear coding to detect multiple abnormalities in WCE frames. Anjany and Surya,^19^ extracted speed‐up robust features (SURF) which are used for classification. Lack of distinguish pattern and manually crafting of a feature vector of SURF, the author used a convolutional neural network to learn texture feature from various abnormal endoscopic findings.

Pixel‐level methods are supposed to be more accurate in order to classify bleeding and nonbleeding pixel samples efficiently. Yuan^20^ extracted color features on the pixels in WCE frames and used thresholding in the color space to identify bleeding regions. Jia^21^ presented an automated bleeding detection strategy which includes discrimination of the bleeding and nonbleeding frames, and, later, applying segmentation on the bleeding region using pattern recognition approaches. Moreover, in Ref. 22 the authors used super‐pixel segmentation to reduce the computational complexity with high diagnostic accuracy. In comparison with frame‐level methods, detection using a pixel‐based method is more accurate with respect to high performance and accuracy. However, pixel‐based methods still have a high computational cost, and it is computationally demanding (more than 50,000 frames need to be examined for a single patient).

To summarize, researchers have studied to analyze each and every frame of WCE video sequences to detect the frames with a pathological alteration. These experiments have been performed by using various image processing and pattern recognition techniques to generate proper frame characteristics, for example, computing color and texture features using various color models. These characteristics define the classification on the basis of frame pixels and frame regions for discrimination between normal and abnormal tissue structure. In our previous work, we have developed an algorithm to extract color feature for ulcer using statistical feature analysis.^23^. This work has the contribution to explore color‐ and texture‐dependent features. Most of the techniques extract the color and texture feature from WCE frames. Various methods are dealing with the individual pixel value, although the blocks of pixels have the potential to detect bleeding frames with high‐performance metrics such as sensitivity, specificity, and accuracy.

## 3 BLEEDING DETECTION

**Figure 3:**
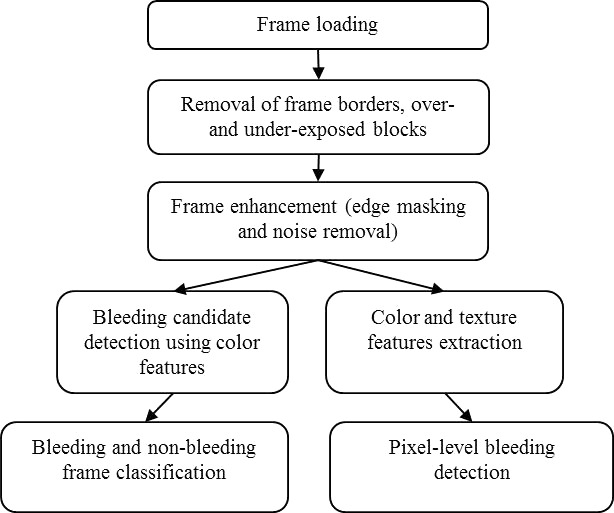
The frame processing sequence of the proposed bleeding detection method.

The bleeding detection technique proposed in this paper is shown in Fig. 3. It was done in two phases, where the classification of bleeding and nonbleeding frame is performed in the first phase using only color features and classification of bleeding and nonbleeding pixel is performed in the second phase using color and texture features. First, we perform an input frame loading with an appropriate data format conversion to RGB color space. Then, we perform a removal the frame borders, over‐ and under‐exposed pixel block to reduce the number of fault detection in these areas. Next, a frame enhancement step is performed using edge masking and noise removal. Later, the feature extraction and bleeding detection are performed using color and texture features. Finally, a classification step is performed for bleeding detection at a frame‐level and a pixel‐level with an appropriate classifier.

### 3.1 Removal of bright and dark blocks

In the GI tract, there can be areas that are both under‐ and over‐illuminated, and which are, therefore, cannot be processed. For example, a large air bubble packet^23^ can be categorized in this class. Luminance is computed as the square root of a sum of individual RGB squares. We have calculated this for each 16x16 block of pixels:

(1) $$I_{\text{Block}}\left(i,j\right)=\sqrt{R_{\text{Block}}^{2}\left(i,j\right)+G_{\text{Block}}^{2}\left(i,j\right)+B_{\text{Block}}^{2}\left(i,j\right)},$$

where *i* and *j* are the horizontal and vertical indices in the frame, respectively.

### 3.2 Edge and noise removal

We encounter various false results due to the presence of edge information in frames which may lead to a wrong detection. These edges are basically intestinal folds, and the ambiguous edging is caused by a random vector of camera’s view direction. To eliminate this information, we are using a canny operator.^24^ The parameters of the canny algorithm allow recognizing edge with differing characteristic depending on desired requirements. For this experimental setup, we have chosen a standard deviation of 0.35% and 35% of the pixels in the frame to reduce noise and to perform a robust detection of pixels at the edges. Hence, we have used the upper and lower threshold values of *τ_1_* = 0.3 and *τ_2_* = 0.7, respectively. Morphological dilation is then applied to dilate the detected information. If *A* is a frame after masking operation and *B* is the structural element, then dilation of *A* by *B* is defined as follows^22^:

(2) $$A⊕B=\left\{z|\left(\hat{B}\right)\;z\;\cap \;A\;\neq \;\emptyset \right\}$$

**Figure 4:**
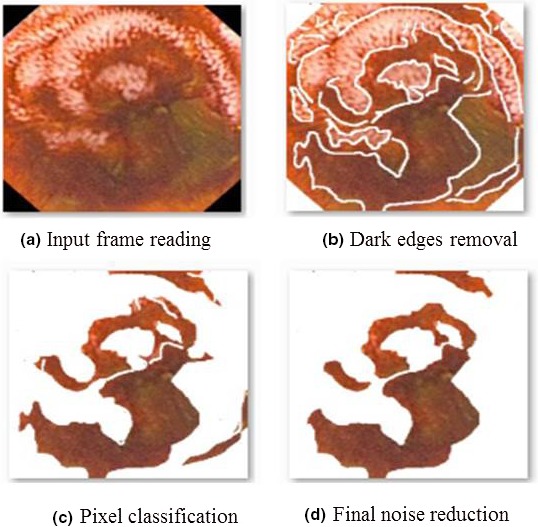
The example of the frame processing steps output for frame‐level bleeding detection procedure.

The above equation is based on getting the reflection of *B* to its origin, and the reflection is shifted by *z*. For this experiment, we have chosen the structuring element of dilation *B* to be a square with a three‐pixel width. Morphological erosion is performed later to remove few bleeding pixels wrongly detected to enhance final result [Fig. 4(d)].

Also, frame enhancement^25^ is required to highlight key data by removing auxiliary information in a frame. We have removed any Gaussian noise using wavelet denoising with three levels of decomposition. Wavelet db2 with soft thresholding is applied to reduce noise and enhance relevant information in bleeding frames.

### 3.3 Color features

Color is one of the most often used features of images, and it can be specified by using various color models. Once the color space is defined, the color feature can be extracted from the frame or a particular defined region. In the RGB color space, the optical frequency bands are defined as 630–780 nm for red (R) band, 490–560 nm for green (G) band, and 450–490 nm for blue (B). For bleeding, the red channel has a high reflectivity, but the green and blue channels have comparatively lower reflectivity and a little difference between values. Thus, we can detect a bleeding region by detecting high red areas, and by computing the red ratio feature for individual pixels containing the three components as features *C*1, *C*2, and *C*3 are shown in eqs. (3)–(5), respectively. The *C*3 feature is the proportion of the R channel in all three primary colors which is also called the chromaticity. Our fourth feature (*C*4) is the ratio of the red channel with the vector amplitude of the green and blue channels as represented in eq. (6). The chroma value for bleeding is very high compared to normal mucosa, and the chroma value is therefore used as another feature (*C*5), as shown in eq. (7).

(3) $$C1=\frac{R\left(i,j\right)}{G\left(i,j\right)}$$

(4) $$C2=\frac{R\left(i,j\right)}{B\left(i,j\right)}$$

(5) $$C3=\frac{R\left(i,j\right)}{R\left(i,j\right)+G\left(i,j\right)+B\left(i,j\right)}$$

(6) $$C4=\frac{R\left(i,j\right)}{\sqrt{G_{\text{Block}}^{2}\left(i,j\right)+B_{\text{Block}}^{2}\left(i,j\right)}}$$

(7) $$C5=1-\frac{\min\left(G\left(i,j\right),B\left(i,j\right)\right)}{R\left(i,j\right)}$$

### 3.4 Color‐based classification

The color features extracted are then used as an input to SVM supervised learning model. SVMs are accurate as they contain appropriate kernels (implicit mappers of inputs into high‐dimensional feature spaces) that work well even if the data are not linearly separable in the future base space. By using the kernel functions of SVMs,^24^ one can perform a nonlinear classification more accurately by mapping its input to high‐dimensional feature spaces.^25^ Various hyper‐planes separate the input instances between a set of predefined classes (two in our use‐case). However, it is important to select the best one which has the largest distance to the nearest data point of two classes. Grid search^25^ is the conventional method of performing the optimization of hyper‐parameter utilizing parameter sweep or grid search through a manually specified subset of the hyper‐parameter of a learning algorithm. This algorithm must be guided by some performance metric, normally measured by evaluation on a held‐out validation set or by cross‐validation of the training dataset. In this article, we are using an SVM classifier with a radial basis function (RBF) kernel having at least two parameters (regularization constant C and kernel hyperparameter γ) that need to be tuned to achieve high performance on the testing data. The mathematical descriptor is shown below for a binary classification problem: {(*x*_1_, *y*_1_), (*x*_2_, *y*_2_), …, (*x_k_*, *y_k_*)}, where *x_i_ ϵ* R*_n_* represents the *n*‐dimensional feature vectors, and *y_i_*_ ϵ_ {1, −1} is the corresponding class label. The SVM requires the solution of the following optimizing problem:

(8) $$\min\left(\frac{1}{2}\omega^{T}\omega +C\sum_{i=1}^{k}\varepsilon i\right),$$

$$\text{subject to}\;y_{i}\left(\omega^{T}\phi \left(x_{i}\right)+b\right)\geq 1-\varepsilon_{i},\;\varepsilon_{i}\geq 0,\;i=1,\ldots ,k.$$

here, *ε_i_*is the slack variable for misclassified examples, and C is the penalty parameter of the error term. In addition, *K*(*x_i_*, *x_j_*) = *φ*(*x_i_*)*^T^* *φ*(*x_j_*) is the kernel function. There are four kernel functions used for the pattern recognition and classification: a linear kernel, a polynomial kernel, an RBF and a sigmoid kernel. We have adopted the RBF^24^ kernel in this paper:

(9) $$K\left(x_{i},x_{j}\right)=\exp\left(-\gamma \|x_{i}-x_{j}{\|}^{2}\right),\;\gamma >0.$$

here, *γ* is the parameter which must be carefully selected in the experiment. The optimum values for the parameter *C* and log_2_ *γ* were selected from the range: (−8, 7, 6, …, 6, 7, 8). The grid method^25^ was adopted as the searching procedure (a 0.8 step was used). Each *γ* and *C* value pair was used in the training data with tenfold cross‐validation in order to evaluate the model performance. Once the optimal values of *γ* and *C* were found, they were adopted to train a new SVM model.

The feature vector used as an input for our SVM‐based detection approach is defined as [*C*1, *C*2, *C*3, *C*4, *C*5]. After removal of dark spots, as shown in Fig. 3(b), each pixel is classified as either bleeding or nonbleeding pixels. All the features are fed to SVM which considers three types of kernels, that is, polynomial, linear, and RBF. The number of pixels is considered as the threshold for frame classification which depicts whether the current frame is showing bleeding or nonbleeding areas. A frame containing bleeding pixels is labeled as a bleeding sample; otherwise, it is labeled as a negative sample.

### 3.5 Texture features

Texture is a very useful feature for a wide range of use cases in image processing and classification tasks. It is generally assumed that the human visual system uses textures for recognition and interpretation of visual input. In general, color is usually a pixel property while texture can only be measured from a group of pixels.^26^ A large number of techniques have been proposed^27^ to extract texture features. Based on the domain from which the texture feature is extracted, they can be broadly classified into spatial texture feature extraction methods and spectral texture feature extraction methods. For the former approach, texture features are extracted by computing the pixel statistics or finding the local pixel structures in the original frame domain, whereas the latter transforms a frame into a frequency domain and then calculates features from the transformed frame. Spatial texture features can extract information from any shape without loss of data but are sensitive to noise and distortion. Spectral texture features are robust and need less computation power, but have no semantic meaning and need square frame regions of sufficient size for extraction.

One of the good candidates for the texture analysis is a statistical method of examining texture that considers the spatial relationship of pixels is the gray‐level co‐occurrence matrix (GLCM). GLCM is a matrix that is defined over a frame to be the distribution of co‐occurring pixel values (grayscale values, or colors) at a given offset, also known as the gray‐level spatial dependence matrix. The GLCM functions characterize the texture of a frame by calculating how often pairs of pixel with specific values and in a specified spatial relationship occur in a frame, creating a GLCM, and then extracting statistical measures from this matrix.^27^ The horizontal direction 0^°^ with a range of 1 (nearest neighbor) was used in this work. The 22 texture descriptions extracted from each of the gray tone spatial dependence matrices are presented in Table 1. The following pre‐defined formulas are used:

(10) $$p\left(i,j\right)=\frac{P\left(i,j\right)}{R},$$

(11) $$p_{x}\left(i\right)=\sum_{j=1}^{\mathrm{Ng}}P\left(i,j\right),$$

**Table 1:** Used texture features.

Feature identifier	Feature	Definition
T1	ASM^28^	$T1=\sum_{i=1}^{\mathrm{Ng}}\sum_{j=1}^{\mathrm{Ng}}{\left\{p\left(i,j\right)\right\}}^{2}$
T2	Entropy^28^	$T2=-\sum_{i=1}^{\mathrm{Ng}}\sum_{j=1}^{\mathrm{Ng}}p\left(i,j\right)\log\left(p\left(i,j\right)\right)$
T3	Dissimilarity^28^	$T3=\sum_{i=1}^{\mathrm{Ng}}\sum_{j=1}^{\mathrm{Ng}}\left|i-j\right|p\left(i,j\right)$
T4	Contrast^28^	$T4=\sum_{n=0}^{Ng-1}n^{2}\left\{\sum_{i=1}^{\mathrm{Ng}}\sum_{j=1}^{\mathrm{Ng}}p\left(i,j\right)\left|i-j\right|=n\right\}$
T5	Inverse difference^28^	$T5=\sum_{i=1}^{\mathrm{Ng}}\sum_{j=1}^{\mathrm{Ng}}\frac{1}{1+\left|i-j\right|}p\left(i,j\right)$
T6	IDM^28^	$T6=\sum_{i=1}^{\mathrm{Ng}}\sum_{j=1}^{\mathrm{Ng}}\frac{1}{1+\left({\left(i-j\right)}^{2}\right)}p\left(i,j\right)$
T7	Correlation^28^	$T7=\frac{\sum_{i=1}^{\mathrm{Ng}}\sum_{j=1}^{\mathrm{Ng}}\left(\text{ij}\right)p\left(i,j\right)-\mu_{x}\mu_{y}}{\sigma_{x}\sigma_{y}}$
T8	Autocorrelation^28^	$T8=\sum_{i=1}^{\mathrm{Ng}}\sum_{j=1}^{\mathrm{Ng}}\left(\text{ij}\right)p\left(i,j\right)$
T9	Cluster shade^28^	$T9=\sum_{i=1}^{\mathrm{Ng}}\sum_{j=1}^{\mathrm{Ng}}{\left(i+j-\mu_{x}-\mu_{y}\right)}^{3}p\left(i,j\right)$ 0
T10	Cluster prominence^28^	$T10=\sum_{i=1}^{\mathrm{Ng}}\sum_{j=1}^{\mathrm{Ng}}{\left(i+j-\mu_{x}-\mu_{y}\right)}^{4}p\left(i,j\right)$
T11	Maximum probability^28^	$T11=\max_{i,j}p\left(i,j\right)$
T12	Variance^28^	$T12=\sum_{i=1}^{\mathrm{Ng}}\sum_{j=1}^{\mathrm{Ng}}{\left(i-\mu \right)}^{2}p\left(i,j\right)$
T13	Sum average^28^	$T13=\sum_{i=2}^{2Ng}{\text{ip}}_{x+y}\left(i\right)$
T14	Sum variance^28^	$T14=\sum_{i=2}^{2Ng}{\left(i-T15\right)}^{2}p_{x+y}\left(i\right)$
T15	Sum entropy^28^	$T15=-\sum_{i=2}^{2Ng}p_{x+y}\left(i\right)\log\left\{p_{x+y}\left(i\right)\right\}$
T16	Difference variance^28^	$T16=\text{variance}\;\text{of}\;p_{x-y}$
T17	Difference entropy^28^	$T17=-\sum_{i=2}^{2Ng}p_{x+y}\left(i\right)\log\left\{P_{x-y}\left(i\right)\right\}$
T18	IMC1^28^	$T18=\frac{\text{HXY}-\text{HXY}1}{\max\left\{\text{HX},\text{HY}\right\}}$
T19	IMC2^28^	$T19={\left(1-\exp\left[-2.0\left(\text{HXY}2-\text{HXY}\right)\right]\right)}^{\frac{1}{2}}$
T20	Maximal correlation coefficient^28^	$T20={\left(\text{Second}\;\text{largest}\;\text{eigen}\;\text{value}\;\text{of}\;\text{the}\;\text{matrix}\;Q\right)}^{1/2}$
T21	INN^28^	$T21=\sum_{i=1}^{\mathrm{Ng}}\sum_{j=1}^{\mathrm{Ng}}\frac{p\left(i,j\right)}{1+{\left|i-j\right|}^{2}/{\mathrm{Ng}}^{2}}$
T22	IDN^28^	$T22=\sum_{i=1}^{\mathrm{Ng}}\sum_{j=1}^{\mathrm{Ng}}\frac{p\left(i,j\right)}{1+{\left(i-j\right)}^{2}/{\mathrm{Ng}}^{2}}$

ASM, angular second moment; IDM, Inverse difference moment; ICM, Information measures of correlation; INN, Inverse difference normalized; IDN, Inverse difference moment normalized.

(12) $$p_{y}\left(i\right)=\sum_{i=1}^{\mathrm{Ng}}P\left(i,j\right),$$

(13) $$p_{x+y}\left(k\right)=\sum_{i=1}^{\mathrm{Ng}}\sum_{j=1}^{\mathrm{Ng}}p\left(i,j\right)\left|i+j=k,k=2,3\ldots ,2Ng\right.,$$

(14) $$p_{x-y}\left(k\right)=\sum_{i=1}^{\mathrm{Ng}}\sum_{j=1}^{\mathrm{Ng}}p\left(i,j\right)\left|\left|i-j\right|=k,k=0,1,\ldots ,Ng-1\right.,$$

(15) $$\text{HXY}=-\sum_{i=1}^{\mathrm{Ng}}\sum_{j=1}^{\mathrm{Ng}}p\left(i,j\right)\log\left(p\left(i,j\right)\right),$$

(16) $$\text{HXY}1=-\sum_{i=1}^{\mathrm{Ng}}\sum_{j=1}^{\mathrm{Ng}}p\left(i,j\right)\log\left\{p_{x}\left(i\right)p_{y}\left(j\right)\right\},$$

(17) $$\text{HXY}2=-\sum_{i=1}^{\mathrm{Ng}}\sum_{j=1}^{\mathrm{Ng}}p_{x}\left(i\right)p_{y}\left(j\right)\log\left\{p_{x}\left(i\right)p_{y}\left(j\right)\right\},$$

(18) $$Q\left(i,j\right)=\sum_{k}\frac{p\left(i,k\right)p\left(j,k\right)}{p_{x}\left(i\right)p_{y}\left(j\right)},$$

where *p(i, j)* is the *(i, j)‐th* entry in a normalized gray‐tone spatial‐dependence matrices, *p_x_(i)* is the *i‐th* entry in the marginal probability matrix obtained by summing the rows of *p(i, j), N_g_* is the number of distinct gray levels in the quantized frame, and *HX* and *HY* are the entropies of *p_x_* and *p_y_*.

The entire textural features are extracted from the gray‐tone spatial‐dependence matrices. The equations, which define a set of 22 measures of textural features, are presented in Table 1. The mentioned features T1, T2, T5, T6, T12‐T19 are taken from the Haralick feature,^27^ the features T3, T8‐T11 are inspired from,^28^ and the other features T5, T21, and T22 are used from 29.

The feature T1 is also called Energy or Uniformity, which is a measure of homogeneity of a frame. A similar scene will contain only a few gray levels, giving a GLCM with only a few but relatively high values of *P(i, j)*. Thus, the sum of squares will be high.

T2 is the entropy function, which is the randomness or the degree of disorder present in the frame. The value of entropy is significant when all elements of the co‐occurrence matrix are the same and small when elements are unequal. Inhomogeneous scenes have low first order entropy, while a similar scene has high entropy. In dissimilarity, (T3) the weights with which GLCM probabilities are multiplied, increase linearly away from the diagonal (along which neighboring values are equal).

The features T3‐T6, T18, and T19 are the smoothness statistics, which use a weighted distance from the main diagonal of the GLCM (i.e., location.). The inverse difference moment (IDM) (T6) is also called homogeneity, and it measures the local homogeneity of a frame. The IDM feature obtains the measures of the closeness of the distribution of the GLCM elements to the GLCM diagonal. IDM has a range of values to determine whether the frame is textured or nontextured. Homogeneity measures how close the distribution of elements in the GLCM is to the diagonal of GLCM. As homogeneity increases, the contrast, typically, decreases.

The correlation (T7) feature measures how correlated a pixel is to its neighborhood. Correlation is a measure of gray level linear dependence between the pixels at the specified positions relative to each other. Feature values range from −1 to 1, these extremes indicating perfect negative and positive correlation, respectively. The $\mu_{x},\mu_{y},\sigma_{x},\;\text{and}\;\sigma_{y}$ parameters/values are the mean and standard deviation of *P_x_* and *P_y_*_. _If the frame has horizontal textures, the correlation in the direction of 0*° *degree is often more significant than those in other directions.

The cluster shade (T9) is a measure of the skewness of the matrix and catches the perceptual concepts of uniformity 30 and works as follows: A new “*i + j*” frame is created, having a range of integer intensities from 0 to 2(*N_g_* − 1). The *u_i + j_* value is computed and stored for the first neighborhood of the frame and is subsequently updated as the neighborhood is moved by one pixel. When the cluster shade is high, the frame is asymmetric.

Cluster prominence (T10) is also a measure of asymmetry.^30^ When the cluster prominence value is high, the frame is less symmetric. In addition, when the cluster prominence value is low, there is a peak in the GLCM matrix around the mean values. For an ultrasound image, a low cluster prominence value indicates small variation in gray‐scale. Maximum probability (T11) is the simple static records in the central pixel of the window, the most significant *p(i, j)* value found within the window. High maximum probability values occur if one combination of pixels dominates the pixel pairs in the window.

Variance (T12) is also known as Sum of Squares and is a measurement of heterogeneity which is strongly correlated to first order statistical variable such as standard deviation. The variance increases when the gray level values differ from their mean. It also includes the average calculated over the sum of adjacent pixels (T13), the variance calculated over the sum of the adjacent pixels (T14), the variance over the difference between adjacent pixels (T16), the entropy on the sum of the adjacent pixels (T15) and the entropy on the difference of the adjacent pixel (T17).

The informational coefficient of correlation (T18 and T19) is a function of the joint probability density function p(x, y) of the two variables *x* and *y*. It is an invariant under a change of parameterization *x’* = *f(x)*, *y’* = *f(y)*, and reduces to the classical correlation coefficient when *p(x, y)* is normal.

The maximal correlation coefficient (T20) defines the square root of the second largest Eigenvalues of the matrix *Q*. It expresses the singular value characterization for the finite‐valued random variables.

### 3.6 A combined color‐ and texture‐based classification

The color and texture features described earlier are designed to extract the different local characteristics at a level of individual pixels. In this work, we are using the machine‐learning‐based two‐class‐classification methods to differentiate between two different pixel groups. The first group includes all pixels that show bleeding‐related findings, like fresh and old blood, open wounds, etc. These findings are typically colored in shades of red: from bright red for fresh blood to dark brown for old blood residuals. At first glance,^30^ the color features seem to be the best option for the detection of areas with bleeding, but, in general, it is not true because of the second group of pixels which are dominating in the GI tract frames.

The second group of pixels is associated with different findings that are not‐bleeding‐related, like normal GI tract tissue, stool masses, food leftovers, bubbles, instruments, water, over‐ under‐illuminated areas, etc. All these “normal” findings can be colored in more or less random colors, but they can also be colored in shades of red, for example, food leftovers, some types of fecal masses, some types of normal GI tract tissue, etc. In contrast to the first group of pixels, for this group of pixels, the texture is an important characteristic that allows the system to distinguish between different types of findings, and in combination with the color information, the texture‐related local characteristics are an essential input for machine‐learning methods to perform pixel‐level classification. Detailed pixel‐perfect classification is essential because the bleeding areas can be both big and very small and, at the same time, the state‐of‐the‐art WCE devices have a relatively low spatial resolution. Thus, an evaluation of each pixel is essential for high‐performance bleeding detection in this scenario.

In the proposed pixel‐level classification approach, we use Random Tree (RT),^30^ Random Forest (RF),^30^ and Logistic Model Tree (LMT)^30^ machine‐learning‐based classifiers with the input vectors consist of the different combinations of the color and the texture features.

## 4 EXPERIMENTAL RESULTS

**Figure 5:**
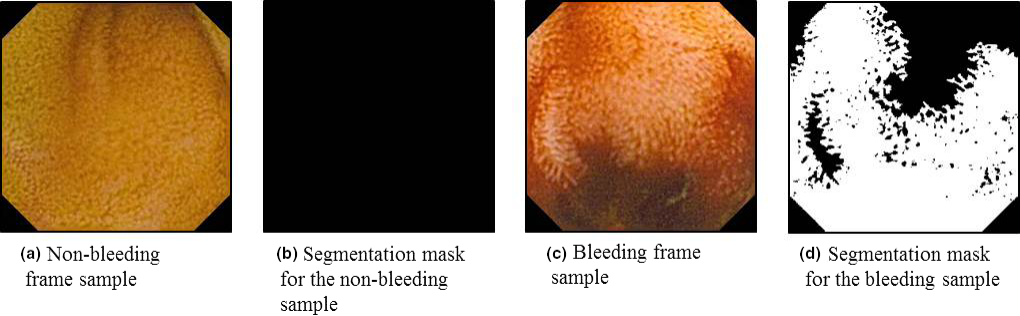
The sample wireless capsule endoscopy frames and their segmentation masks.

This research has obtained its novelty through collaboration with the medical experts (experienced endoscopists) from Endoscopy unit at University of Malay Medical Center (UMMC), Kuala‐Lumpur, Malaysia. The data collection has been performed at their medical center especially to carry out this research. In order to validate our results, the experts provided the ground truth data in the form of frame labels (bleeding/nonbleeding) for all the collected frames and the pixel‐level bleeding areas segmentation masks [see Fig. 5(d) for example] for bleeding frames only with the highlighted bleeding areas. The WCE devices used to record the frames were Olympus Endocapsule with the resolution of 288 × 288 pixels per frame.

### 4.1 Performance metrics

The performance metrics used in the experimental evaluation of our methods are accuracy (ACC), precision (PREC), sensitivity or recall (REC), specificity (SPEC), F‐Measure (F1), Matthews correlation coefficient (MCC), area under receiver operator characteristic curve^30^ and area under precision‐recall curve.^30^ Four cases can be recorded for detection of bleeding and nonbleeding frames and pixels. A bleeding frame (pixel) which is detected as a nonbleeding frame is called false nonbleeding detection or false negative (FN). A nonbleeding frame (pixel) which is detected as a bleeding frame is called a false bleeding detection or false positive (FN). The other two cases are true bleeding detection or true positive (TP) and true nonbleeding recognition or true negative (TN). The performance metrics are defined as the following:

(19) $$\text{Accuracy}=\frac{\text{TP}+\text{TN}}{\text{TP}+\text{FP}+\text{TN}+\text{FN}}$$

(20) $$\text{Precision}=\frac{\text{TP}}{\text{TP}+\text{FP}},$$

(21) $$\text{Recall}\;\left(\text{Sensitivity}\right)=\frac{\text{TP}}{\text{TP}+\text{FN}},$$

(22) $$\text{Specificity}=\frac{\text{TN}}{\text{FP}+\text{TN}},$$

(23) $$\text{F-Measure}=\frac{2\text{TP}}{2\text{TP}+\text{FP}+\text{FN}},$$

(24) $$\begin{array}{c} \text{Matthews correlation coefficient}\\ \;=\frac{\text{TP}\times \text{TN}-\text{FP}\times \text{FN}}{\sqrt{\left(\text{TP}+\text{FP}\right)\left(\text{TP}+\text{FN}\right)\left(\text{TN}+\text{FP}\right)\left(\text{TN}+\text{FN}\right)}} \end{array}.$$

For overall performance evaluation of the proposed methods, we selected the MCC metric which is used in machine learning as a measure of the quality of binary (two‐class) classification methods. It takes into account true and false positives and negatives and is generally regarded as a balanced measure that can be used even if the classes are of very different sizes. The MCC value lies in the region between −1 and +1. A coefficient of +1 represents a perfect prediction, 0 no better than random prediction and −1 indicates total disagreement between prediction and observation. Our previous research 31, 32 confirmed that MCC is the most convenient and efficient metric for the binary classification tasks evaluation and comparison.

### 4.2 Frame‐level bleeding detection

As per data availability at UMMC, we have chosen 300 bleeding frames and 200 nonbleeding or normal frames for the training dataset (500 frames). The testing data set consists of 500 bleeding and 200 nonbleeding frames (700 frames). All these samples are randomly extracted from 27 different videos for comparative experiments. All the bleeding frames were annotated by the experienced endoscopists provided the bleeding areas segmentation masks with the true values assigned to the bleeding pixels.

The example output of the sequential frame processing steps is depicted in Fig. 4. The number of pixels that are considered as the positive bleeding detection threshold was set to 280 pixels in order to achieve the optimal bleeding detection performance metrics for this example and all the frame‐level bleeding detection experiments. This threshold value was selected based on our previous studies 33 showed that below this number, the detection method could incorrectly detect angiodysplasia or other small dark patches as a bleeding region.

The experimental results depicted in Table 2 shows the REC, SPEC, ACC, F1, and MCC metrics for the different classifications. Among three different kernels of SVM, RBF kernel shows the best results for classification. Few frames have misclassified as they contained angiodysplasia, which is a small vascular malformation of the gut, also colored in red. The performance of our methods is compared to the best results reported in Ref. 10,24 Although the reported results from other authors also have promising detection results, the proposed methods have less computational costs of 0.38 s per frame due to the simplicity of algorithm in addition to high detection performance.

**Table 2:** Performance comparison with state‐of‐the‐art methods.

Metrics	Method				
	10	24	Proposed method		
			SVM	SVM	SVM
			Polynomial	Linear	RBF
Recall (sensitivity)	0.970	0.931	0.959	0.946	0.976
Specificity	0.936	0.884	0.913	0.917	0.955
Accuracy	0.948	0.915	0.900	0.892	0.977
F1	n/a	n/a	0.962	0.949	0.978
MCC	n/a	n/a	0.868	0.860	0.898

F1, F‐Measure; MCC, Matthews correlation coefficient; SVM, support vector machine.

As we can see from Table 1, we have good results in terms of REC and ACC. Most of the times, the bleeding regions are dark red, and this color is difficult to identify as a bleeding frame. Other bleeding areas in the same frame were well detected as bleeding areas. Therefore, the overall performance of detecting a bleeding frame is high. Specificity is relatively high too considering gastric conditions with the nonuniform light. It might be the case for reduced performance.

### 4.3 Pixel‐level bleedings detection

For the pixel‐level bleeding detection, we have selected a subset of frames from the dataset that has been used for frame‐level bleeding detection experimental studies. First, we have chosen 93 bleeding frames and 186 nonbleeding or normal frames for the pixel‐level detection evaluation data set (279 frames in total). Then, we have randomly divided the selected bleeding and nonbleeding frames into training and test sets containing 47 and 46 bleeding and 93 and 93 nonbleeding frames, respectively. The whole segmentation masks for the nonbleeding frames as well as the not‐bleeding‐related pixels in areas in bleeding frames, like normal GI tract tissue, stool masses, parasites, food leftovers, bubbles, instruments, water, over‐ and under‐illuminated areas, frame borders, etc. are marked with false values. The examples of the source frames and the corresponding segmentation masks are depicted in Fig. 5.

The different color and texture features can provide different amounts and quality of information. Regardless of the native properties of the machine‐learning methods, which can support automatic selection of the most meaningful features, it is essential to understand and be able to estimate the importance of the different color and texture features. In this research, we have performed a simple analysis of the color and texture features in terms of the value for the binary bleeding classification. During this analysis, we extracted all the color and texture features from all the frames in both the training and test sets. The features extracted then were used in tenfold cross‐validation of a single‐feature‐based RT classifier.

Table 3 depicts a visual representation of the extracted color and texture features for the two sample frames (see Fig. 5) and the corresponding weighted average of the MCC measure values for bleeding classification performance. As one can see, all the color and texture features outperform the ZeroR base‐line classifier with zero MCC value. All the individual color features showed the promising performance with MCC values from 0.449 up to 0.832. Thus, all the color features can be considered as the right candidates for the pixel‐level bleeding detection. The individual texture features shown the MCC value varies in a range from 0.151 to 0.486 that is significantly lower than the color features. However, the MCC values greater than zero correspond to valid and better‐than‐random predictions. Thus, we can expect small but noticeable benefits of combining color and texture features in one feature vector. For the following experiments, we have therefore selected all the color features as a joint feature vector base (C1‐C5). The texture features had been used in different combinations which included (a) all the texture features (T1‐T22), (b) the top‐MCC texture features (T4, T6, T8, T13 and T14), and (c) the visually different texture features with the highest possible MCC (T4, T6, T9, T11, and T13).

**Table 3:**
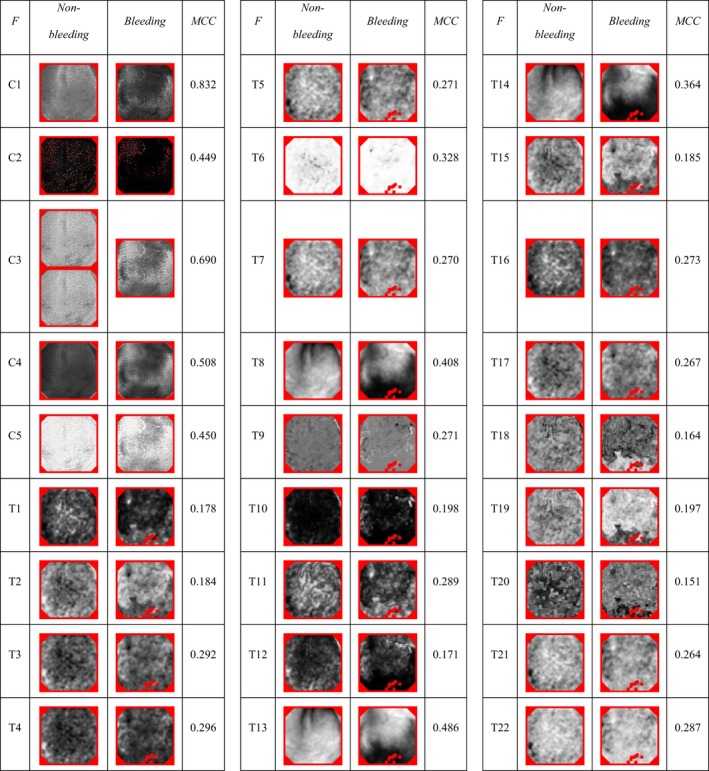
The comparison of the color and texture features (marked as “F”) for the bleeding (marked as “Bleeding”) and normal (marked as “Nonbleeding”) WCE samples with the corresponding bleeding pixels detection tenfold cross‐validation weighted average MCC performance. The color and texture features are marked with “C” and “T” prefixes respectively with the following texture identifier. All the feature output frames are range‐normalized. Red color is used to mark pixels that are nonmeaningful or contain nonnumbers after the features extraction.

In the following experiments, we have evaluated the different approaches to the pixel‐level bleeding detection using the different combinations of the color and texture features and the different variants of the machine‐learning methods used for pixel classification. The feature combinations used include the five best‐performing color features only, the five best‐performing texture features only and three combinations of all the color and different texture features: [T1‐T22], [T4, T6, T8, T13, T14], and [T4, T6, T9, T11, T13] named "All", "Five top", and "Five different" respectively in the following tables. All the color and texture features were extracted for each meaningful pixel (not a black frame border, not an over‐ and under‐exposed pixel) of the input frames using the corresponding equations described in sections 3.4 and 3.5. The features extracted were combined using the simple early‐fusion approach when the resulting pixel's feature vector made as a simple enumeration of all the feature values used. The machine‐learning approaches used are RT, RF, and LMT; all have been proven 31, 32, 34 to be able to provide a good two‐class classification performance for GI tract frames. RT is the simplest one that therefore has the lowest computation complexity with good enough performance. RF and LMT are balanced in terms of complexity and the performance provided, while RF is more straightforward to compute and LMT has a slightly better performance depending on the data being classified.

Table 4 depicts the results of the tenfold cross‐validation using the entire pixel‐level bleeding detection dataset which consist of 93 bleeding and 186 nonbleeding frames. As one can see, all the proposed combinations of features and machine‐learning‐based classifiers can significantly outperform the base‐level ZeroR classifier. As it was expected form the individual features performance evaluation, the texture‐only run with RT classifier had the worst MCC performance value of 0.645. The color‐only run with RT classifier showed much higher performance with an MCC value of 0.824. The significantly higher performance of the color‐only‐based approach is expected due to a primary color‐based nature of bleedings. The following runs were performed using the combination of all the color features and different texture features in order to verify the theory of the potential advantages of texture information for bleeding detection. The total number of the combined runs were evaluated is 9–3 runs per the classifier. As it was expected from our previous experience 32, RT classifier performs the worst in terms of classification performance comparing to RF and LMT resulting in the highest MCC performance of 0.895 for all the color and [T4, T6, T9, T11, T13] texture features. The RF and LMT classifiers achieved the comparable classification performance. Controversial to our previous experimental studies 32, RF had noticeable better MCC performance of 0.931 for all the color and all the texture features, which is higher comparing LMT with the MCC value of 0.922 for the same feature combination. The relatively low numbers of features and frames (comparing to our previous research) used in this experimental studies can be a reason for such an unexpected behavior of the LMT classifier. Nevertheless, the performance results of this evaluation confirm that proposed combinations of texture and color features provide the significantly better results than color and texture features used alone.

**Table 4:** Tenfold cross‐validation results for the whole pixel‐level bleeding detection dataset.

Color features	Texture features	Classifier	PREC	REC	SPEC	ACC	F1	MCC	ROC	PRC
None	All	RT	0.876	0.875	0.770	0.875	0.876	0.645	0.823	0.836
All	None	RT	0.938	0.938	0.883	0.938	0.938	0.824	0.911	0.912
All	All	RT	0.962	0.962	0.930	0.962	0.962	0.893	0.946	0.946
All	Five top	RT	0.963	0.963	0.931	0.963	0.963	0.894	0.947	0.946
All	Five different	RT	0.963	0.963	0.932	0.963	0.963	0.895	0.947	0.947
All	All	RF	0.976	0.976	0.959	0.976	0.976	0.931	0.997	0.997
All	Five top	RF	0.975	0.974	0.956	0.974	0.975	0.928	0.996	0.996
All	Five different	RF	0.976	0.975	0.958	0.975	0.975	0.930	0.997	0.996
All	All	LMT	0.973	0.973	0.954	0.973	0.973	0.922	0.995	0.994
All	Five top	LMT	0.970	0.970	0.945	0.970	0.970	0.914	0.994	0.993
All	Five different	LMT	0.973	0.972	0.954	0.972	0.972	0.922	0.995	0.994
‐	‐	ZeroR	0.598	0.773	0.227	0.773	0.674	0.000	0.500	0.649

PREC, precision; REC, sensitivity or recall; SPEC, specificity; F1, F‐Measure; ACC, accuracy; MCC, Matthews correlation coefficient; ROC, receiver operator characteristic curve; PRC, precision‐recall curve; RT, Random tree; LMT, logistic model tree.

To investigate the validity and potential of the proposed feature combinations for the real‐world bleeding detection approaches, we have performed twofold cross‐validation of the algorithms using the previously created training and test datasets. The validation results are depicted in Table 5. The detailed analysis of the performance numbers confirmed the already discovered interrelationships in the performance of different runs regarding the sets of features and the machine‐learning approach used. The RF classifier with the combination of all the color and all the texture features performed best with an MCC score of 0.903. Moreover, the measured difference between all the performance metrics measured during the original‐ and flipped‐order runs is small (with the maximum value of 0.014 for MCC measure) and shows a slightly better detection performance for the original order of training and test sets for all the runs, what is confirm is that the bleeding detection approach is complete, valid, and can be used in real‐world applications with the real datasets obtained from WCE endoscopic procedures.

**Table 5:** Twofold cross‐validation results for the two pixel‐level bleeding detection datasets. The performance measures are presented in the original/ flipped order regarding the selected training and test sets.

Color features	Texture features	Classifier	PREC	REC	SPEC	ACC	F1	MCC	ROC	PRC
None	All	RT	0.882/0.851	0.875/0.854	0.762/0.745	0.875/0.854	0.878/0.852	0.607/0.620	0.818/0.800	0.850/0.800
All	None	RT	0.936/0.921	0.934/0.922	0.873/0.864	0.934/0.922	0.935/0.922	0.787/0.800	0.903/0.893	0.914/0.887
All	All	RT	0.958/0.943	0.957/0.944	0.911/0.898	0.957/0.944	0.957/0.943	0.859/0.856	0.934/0.921	0.941/0.916
All	Five top	RT	0.959/0.944	0.958/0.944	0.918/0.896	0.958/0.944	0.958/0.944	0.863/0.857	0.938/0.920	0.943/0.916
All	Five different	RT	0.958/0.946	0.958/0.946	0.912/0.903	0.958/0.946	0.958/0.946	0.861/0.862	0.935/0.925	0.941/0.920
All	All	RF	0.971/0.957	0.970/0.957	0.946/0.924	0.970/0.957	0.971/0.957	0.903/0.890	0.993/0.985	0.992/0.985
All	Five top	RF	0.970/0.955	0.969/0.955	0.945/0.919	0.969/0.955	0.969/0.955	0.899/0.886	0.992/0.984	0.990/0.984
All	Five different	RF	0.971/0.956	0.970/0.956	0.945/0.922	0.970/0.956	0.970/0.956	0.902/0.888	0.993/0.984	0.991/0.984
All	All	LMT	0.968/0.955	0.968/0.956	0.940/0.923	0.968/0.956	0.968/0.955	0.894/0.887	0.989/0.982	0.984/0.983
All	Five top	LMT	0.969/0.954	0.968/0.955	0.944/0.918	0.968/0.955	0.968/0.954	0.896/0.884	0.988/0.982	0.979/0.981
All	Five different	LMT	0.968/0.954	0.967/0.954	0.939/0.920	0.967/0.954	0.967/0.954	0.893/0.883	0.988/0.983	0.977/0.983
‐	‐	ZeroR	0.665/0.533	0.816/0.730	0.184/0.270	0.816/0.730	0.733/0.616	0.000/0.000	0.500/0.500	0.699/0.605

PREC, precision; REC, sensitivity or recall; SPEC, specificity; F1, F‐Measure; ACC, accuracy; MCC, Matthews correlation coefficient; ROC, receiver operator characteristic curve; PRC, precision‐recall curve; RT, Random tree; LMT, logistic model tree.

However, the measured difference between the best performing RF‐based bleeding detection runs with all the texture, and the [T4, T6, T9, T11, T13] texture features is almost nonnoticeable with the difference value of 0.001, thus using of only these five texture features in combination with five color features recommended for reducing the classification computation complexity. In our future work, after collecting and annotating of a more significant number of bleeding frames form a broader range of patients and bleeding cases, we are going to re‐evaluate the value of the different texture features using the statistical methods in order to find the combination of texture features with the best possible performance and computational complexity balance for this use‐case.

A comparison of our bleeding detection method to the best state‐of‐the‐art methods is depicted in Table 6. The direct comparison of our method to the existing methods is difficult because the different research teams are reporting different and sometimes nonoverlapping performance metrics. However, the obtained results from the related work performance numbers allow performing the comparison based on PREC, REC, specificity, accuracy, and F‐Measure scores. As shown in Table 6, our bleeding pixels detection method outperforms the Refs. 20 and 22 approaches in terms of accuracy achieving accuracy value of 0.976. Next, we perform better in terms of PREC with a value of 0.976 than Ref. 20 and slightly worse than Ref. 21 with the accuracy of 0.99. Sensitivity (recall) of 0.976 achieved in our experiments is better than Refs. 20 and 22, was slightly worse than Ref. 22 with recall value of 0.99. The 0.959 value of specificity is better than results described in Ref. 22 but lower than Ref. 20 with the peak of 0.97. Concerning the F‐Measure score, it is possible to compare our results only to Ref. 21, and we are performing almost as efficient with the F1 value of 0.976 (lower by 0.004).

**Table 6:** Performnce comparison with the state‐of‐the‐art methods.

Bleeding detection method	PREC	REC	SPEC	ACC	F1	MCC	ROC	PRC
20	0.95	0.92	0.97	0.96	n/a	n/a	n/a	n/a
21	0.99	0.97	n/a	n/a	0.98	n/a	n/a	n/a
22	n/a	0.99	0.94	0.95	n/a	n/a	n/a	n/a
Our method	0.976	0.976	0.959	0.976	0.976	0.931	0.997	0.997

PREC, precision; REC, sensitivity or recall; SPEC, specificity; F1, F‐Measure; ACC, accuracy; MCC, Matthews correlation coefficient; ROC, receiver operator characteristic curve; PRC, precision‐recall curve.

Generally speaking, a fair comparison of the different two‐class classification approaches is difficult with the widely used PREC, REC, specificity, accuracy, and even F‐Measure scores because none of these metrics assess the imbalances in the positive and negative samples in the datasets, as well as the sizes of the datasets. In the case of a fully balanced dataset with the equal number of positive and negative samples, the MCC value measured is equal to the F‐Measure value. With the increase of the level of the dataset imbalance, the MCC value becomes lower with the limit of zero for the fully unbalanced dataset. Thereby, comparative analysis of the F‐Measure and MCC score can also be used to estimate the dataset balance for the results obtained using the nonpublic and not well‐described datasets. Thus, the only metrics that can efficiently be used for the direct performance comparison of the different methods on the different datasets is MCC, and we, therefore, invite all the researches to report this metrics or a whole set of TP, TN, FP, and FN values enabling a computation of all the metrics can be used for the method's comparison.

## 5 CONCLUSION

In this paper, we presented a developed automated bleeding detection algorithm that detects the frame with bleeding as well as pixels that are associated with bleeding areas. We briefly describe the related work and the base ideas of our detection approach. We introduced the color and texture features used for frames analysis and presented both our approaches using either color or features and the combined color‐texture‐based approach. The novelty of the best‐performing detection approach includes a combination of the best color and texture features used. A detailed evaluation of the frame‐ and pixel‐level bleeding detection has been performed. The experimental results displayed a good performance of our bleeding detection method in terms detection accuracy at least as good as state‐of‐the‐art approaches. Not only that the novelty of the proposed method promises the higher accuracy, provides a broader comparison of distinctive state‐of‐the‐art features, and various classification methods, alongside with the detection method performance measurement using a comprehensive combination of metrics. The conducted experimental studies confirmed the importance of the features combination even for the relatively simple case of GI tract bleeding detection. Using of both the color and texture features is required for the highest detection performance.

For the future work, we plan to extend the sets of texture and color features used in our classification approach and to perform a more in‐depth statistical analysis of the value of different features for the classification performance. Next, we plan to extend the methods presented in this paper for WCE ulcer frames analysis in order to support UC and inflamed areas detection and localization. Finally, using our previous successful experience^21^ in speeding‐up of feature extraction using heterogeneous resources such as graphical processing units (GPU), we plan to implement the feature extraction code on GPU, which will allow a significant increase in the performance of our proposed detection approach in relevance with frame processing speed.

## CONFLICT OF INTEREST

The authors declare that they have no conflict of interests.
